# What Does It Mean to Be Responsive to a Partner’s Sexual Needs? Toward a Definition of Sexual Need Responsiveness

**DOI:** 10.1007/s10508-022-02432-2

**Published:** 2022-10-12

**Authors:** Laura M. Vowels, Carla A. Roos, Jasmina Mehulić, Siobhan M. O’Dean, M. Dolores Sánchez-Hernández

**Affiliations:** 1 Family and Development Research Center, Institute of Psychology, University of Lausanne, 1015 Lausanne, Switzerland; 2 Department of Social Psychology, Faculty of Behavioral and Social Sciences, University of Groningen, Groningen, The Netherlands; 3 Department of Communication and Cognition, School of Humanities and Digital Sciences, Tilburg University, Tilburg, The Netherlands; 4 Department of Psychology, Faculty of Humanities and Social Sciences, University of Zagreb, Zagreb, Croatia; 5 The Matilda Centre for Research in Mental Health and Substance Use, University of Sydney, Sydney, Australia; 6 University of New South Wales, Sydney, Australia; 7 Department of Social Psychology, Mind, Brain, and Behavior Research Center (CIMCYC), University of Granada, Granada, Spain

**Keywords:** Sexual need responsiveness, Close relationships, Sexual well-being

## Abstract

Being responsive to a partner’s sexual needs in relationships (i.e., sexual need responsiveness) is associated with higher sexual and relationship satisfaction. Previous research has focused on researcher-led definitions of sexual need responsiveness. The purpose of the present study was to develop a participant-informed definition of sexual need responsiveness. A total of 305 individuals responded to five open-ended questions concerning the definition and behaviors pertaining to sexual need responsiveness. The content analysis results showed that the most common elements in participants’ definition included listening to and accommodating each other's sexual needs and wishes whenever possible, while respecting both partners’ safety and boundaries. Both verbal and non-verbal communication in various forms was used to express and share needs with partners and participants often evaluated responsiveness in terms of their sexual satisfaction. The results provide a participant-informed definition of sexual need responsiveness and can be used to inform research and clinical practice.

## Introduction

Today, many partners expect their relationships to provide not only love and nurturance but also a space for self-expansion and growth (Finkel, 2018) as well as fulfilment of sexual needs (Perel, 2017). It is possible for people to meet some of their growth, emotional, and social needs outside of the relationship (Rubin et al., 2014). However, most romantic relationships are ostensibly sexually monogamous (Blanchflower & Oswald, 2004; Levine et al., 2018), which means that most partners rely exclusively on one another for the fulfilment of their sexual needs (Impett et al., 2020). Failure to acknowledge and meet the sexual needs of a partner could result in reduced sexual intimacy, conflict, and dissatisfaction within the relationship, potentially increasing the likelihood of relationship dissolution (Impett et al., 2020). This highlights the importance of partners being sexually responsive within relationships to meet each other’s sexual needs.

Sexual well-being is a core element of most stable and happy romantic relationships (e.g., Birnbaum & Finkel, 2015; Sprecher, 2002). Indeed, there is a growing body of literature that suggests deficits in sexual well-being can be detrimental both within relationships and for the individual. For instance, low sexual well-being has been associated with a decline in overall happiness across 29 countries (Laumann et al., 2006) as well as a decline in relationship satisfaction and increased likelihood of relationship breakup (Mitchell et al., 2013). Many couples struggle to maintain feelings of desire and passion in long-term relationships (for a review, see Impett et al., 2014) and more than one third report experiencing sexual issues including disagreement on frequency of sex or low sexual desire (Miller et al., 2003). Previous research has found that conflicts around sexual issues increase interpersonal vulnerability (e.g., perceiving a conflict as a threat to the self or relationship; Rehman et al., 2017, 2019) and are more predictive of relationship quality than non-sexual conflicts (Rehman et al., 2017). Conflicts around sexual issues are also among the most difficult conflicts to resolve (Geiss & O’Leary, 1981; Sanford, 2003). Taken together, this line of research suggests that being able to express and meet each other’s sexual needs in relationships is particularly important for not only relationship functioning, but also individual well-being.

Being responsive to a partner’s sexual needs (from here on referred to as sexual need responsiveness) may be protective for long-term relationships. For example, previous research has found that individuals who are highly motivated to meet their partner’s sexual needs, experience higher levels of sexual and relationship satisfaction, and relationship commitment compared to individuals who are less motivated to meet their partner’s sexual needs (for reviews, see Impett et al., 2015, 2020; Muise & Impett, 2016). However, what meeting sexual needs means and how people express these needs requires further research. At present, sexual need responsiveness is rarely explicitly defined in the literature and what researchers mean by sexual need responsiveness and how participants interpret the construct may differ. Understanding how participants themselves define sexual need responsiveness can help researchers to measure responsiveness more accurately and more effectively to predict and manipulate sexual need responsiveness. Thus, in the present study, we asked participants to describe what sexual need responsiveness means for them and how they express sexual need responsiveness in their own relationship.

### Perceived Partner Responsiveness

One of the ways in which responsiveness has been measured in the literature in relation to meeting one’s sexual needs is by using perceived partner responsiveness. Perceived partner responsiveness, defined as perceiving a partner as understanding one’s self, validating one’s abilities and opinions, and caring for one’s needs (Reis et al., 2004), is one of the most common constructs studied in relationship research. Perceived partner responsiveness has been found to be an important factor for relationship satisfaction (Reis et al., 2004), and by extension for overall psychological and physical well-being (e.g., Baumeister & Leary, 1995; Uchino et al., 1996). In a sexual context, perceived partner responsiveness has been associated with increased sexual and relationship satisfaction (Rosen et al., 2020) and sexual desire (Birnbaum & Reis, 2012; Birnbaum et al., 2016; van Lankveld et al., 2021). Perceiving one’s partner as responsive has also been shown to aid people in conversations about sex (Merwin & Rosen, 2020). Merwin and Rosen found that perceiving one’s partner as responsive buffered against them engaging in individualistic talk during sex (i.e., self-focused sexual talk relating to one’s own experiences and pleasure, e.g., asking for their needs to be met), having a negative association with sexual and relationship well-being for their partner. Thus, these findings suggest that perceived partner responsiveness is important for partners’ sexual relationship even when it is not explicitly about sex.

In at least one study, perceived partner responsiveness has been adapted to a measure of responsiveness to a partner’s sexual needs (Raposo & Muise, 2021). In the daily diary study of 121 couples, Raposo and Muise used a single item to measure sexual need responsiveness. They examined whether sexual need responsiveness buffered against lower relationship quality of anxiously attached partners and found that on days when anxiously attached individuals perceived their partners as sexually responsive, they also reported higher sexual and relationship satisfaction, commitment, and trust. Thus, these findings suggest that perceiving one’s partner as responsive to one’s sexual needs can be protective for relationship functioning. However, although common in daily diary research, a single item may not fully capture sexual need responsiveness.

### Sexual Communal Strength

Another related construct to sexual need responsiveness is sexual communal strength, which is defined as a desire or willingness to meet a partner's sexual needs (Muise et al., 2013). Communal strength and responsiveness are related but distinct constructs in that communal strength refers to the motivation to meet a partner’s needs, whereas responsiveness reflects a belief in the extent to which one’s partner expresses understanding, care, and validation of these needs (Impett et al., 2020). Individuals high in communal strength are usually perceived as more responsive by their partner and the responsiveness is associated with greater sexual and relationship satisfaction (Muise & Impett, 2015). Sexual communal strength is based on the theory of communal motivation (Clark & Mills, 1979, 2012) which suggests that individuals who are more communally oriented will prioritize satisfying the needs of other people without the expectation of direct reciprocation. In contrast, individuals who are less communally oriented and are more individualistic will prioritize their own needs over their partner’s needs. Interdependence theory suggests that partners become more interdependent as their relationship progresses leading them to become more communally oriented over time (Thibault & Kelley, 1959).

Previous research has found that sexual communal strength is associated with higher sexual and relationship satisfaction (Balzarini et al., 2021; Day et al., 2015; Hogue et al., 2019) and sexual desire (Hogue et al., 2019). For example, Balzarini et al. found across four studies that perceiving partner as falling short in meeting one’s sexual ideals was associated with poorer sexual and relationship satisfaction, but this association was buffered by the partner being high in sexual communal strength in that individuals with partners high in sexual communal strength did not experience this negative association.

The measure of sexual communal strength involves items that focus on placing a high importance on satisfying a partner’s needs. However, the measure also includes an item on sacrificing one’s own needs to satisfy a partner’s needs. As such, sexual communal strength, although positively correlated with higher sexual satisfaction and desire overall, may overlap with a different construct, unmitigated sexual communion (prioritizing a partner’s needs to the exclusion of one’s own needs; Muise et al., 2017, 2018a, 2018b). This may be especially true if people report very high levels of sexual communal strength. Unmitigated sexual communion is associated with lower sexual and relationship satisfaction (Hogue et al., 2019; Impett et al., 2019; Muise et al., 2017, 2018a, 2018b) and lower sexual desire in community samples (Hogue et al., 2019), and in lower sexual functioning and higher levels of pain and anxiety in a clinical sample (Muise et al., 2018a, 2018b). Thus, it is important to discern between the motivation to meet a partner’s sexual needs with a concern for one’s own well-being from the motivation to meet a partner’s sexual needs without a concern for one’s own needs given the drastically different outcomes for sexual communal strength and unmitigated sexual communion. Providing a participant-informed definition can help us understand whether sexual need responsiveness includes an element of prioritizing a partner’s needs above one’s own or not.

### The Present Study

Existing research into responsiveness to sexual needs in relationships has previously been expert-defined, as discussed above. Some definitions have relied on sexual communal strength, which is defined as a motivation to meet a partner’s sexual needs. Alternatively, definitions of responsiveness to sexual needs have relied on perceived partner responsiveness which is not explicitly focused on sexual relationships. In both cases, researchers have provided their definitions of what sexual need responsiveness is (e.g., Impett et al., 2020; Muise & Impett, 2015) rather than participants defining what responsiveness to sexual needs means for them. This approach may conceal aspects of responsiveness that are important to how partnered individuals themselves understand and experience it.

Therefore, the purpose of the present study was to provide a comprehensive definition of sexual need responsiveness based on participants’ responses to a series of open-ended questions. We also aimed to provide a comparison between the definition and how sexual need responsiveness is enacted in relationships. The questions asked participants about how they themselves define sexual need responsiveness, what their experiences of sexual need responsiveness are in relationships, and what behaviors would indicate sexual need responsiveness both as the receiver and provider in a sexual context. We expected that both perceived partner responsiveness and sexual communal strength would be important elements of sexual need responsiveness but there may be other facets that are important for perceiving oneself and one’s partner as responsive to each other’s sexual needs that the existing frameworks do not cover. Specifically, we addressed the following research questions:

1. How do participants define sexual need responsiveness?
2. What are the most commonly described components of sexual need responsiveness?
3. How does the way people define sexual need responsiveness compare to the way they enact it in their relationship?

## Method

### Participants and Procedure

The ethical approval for this study was obtained from University of Southampton. The data were collected in October 2020 using an online survey hosted on the Qualtrics platform. The study was advertised as focusing on “what responsiveness to sexual needs means for you.” We used convenience sampling in which the call to participate was published on the authors’ social networking sites (Twitter and Facebook) and shared by their contacts. Only partnered individuals were eligible for the study because of the focus of the study being on relationship constructs. We also included only participants aged 18 and over. At the beginning of the survey, we asked for participants’ relationship status and age. If the participants reported “single” or said they were under 18, they were directed to the end of the survey. The data were collected as a part of a larger study, which consisted of several questions including socio-demographic information, sexual well-being (Laumann et al., 2006), and sexual need responsiveness for which we adapted the perceived partner responsiveness scale (Reis et al., 2004). In addition, the survey included five open-ended questions pertaining to the definition of and behaviors included in responsiveness to sexual needs. This paper will focus on the results obtained from these five open-ended questions only.

Of the 643 people who took the survey, 338 (52.6%) were excluded because they did not provide answers to any of the open-ended questions (see next section below). Thus, the final sample included 305 individuals (*M*_age_ = 36.34 years, *SD* = 10.76, range 20–68 years; 81.6% women).^1^ The sample was highly educated with most participants having completed at least a master’s degree or Ph.D. (48.5%). Slightly over a half of the sample was employed full-time (59%). The rest were either employed part-time (9.2%), still studying (12.1%), unemployed (4.9%), retired (2.3%) or on parental leave/stay-at-home parent (2.6%). Half of all participants reported being married (49.5%) and almost a third reported being in a committed relationship (28.9%). Almost two-thirds of all participants reported having no children at the time of the survey (62.3%). Most participants were white (87.7%) and half lived in the USA (50.2%). A third of all participants (35.3%) reported having no religion. The average relationship duration was around 9 years (*SD* = 8.44) with a range of 1 month to 40 years. Only 3.3% of respondents reported being in a same sex relationship. Full sample characteristics are shown in Table 1.

**Table 1:** Sociodemographic characteristics of the pooled sample (*N* = 305)

Variable	*n* (%)^a^
*Gender*	
Woman	249 (81.6%)
Man	51 (16.7%)
Other	5 (1.6%)
*Having children*	
Yes	115 (37.7%)
No	190 (62.3%)
*Sexual orientation*	
Heterosexual	216 (70.8%)
Lesbian/gay	10 (3.3%)
Bisexual	71 (23.3%)
Other	7 (2.3%)
Prefer not to say	1 (0.3%)
*Relationship status*	
Dating	10 (3.3%)
In a committed relationship	88 (28.9%)
Cohabiting	56 (18.4%)
Married	151 (49.5%)
*Highest level of completed education*	
Middle school/secondary school	0 (0%)
Some high school/secondary school	0 (0%)
Graduated high school/A-levels	7 (2.3%)
Some college/university	41 (13.4%)
Undergraduate degree	107 (35.1%)
Postgraduate degree (master’s and/or PhD)	148 (48.5%)
Other	2 (0.7%)
*Current employment status*	
Employed full-time	180 (59%)
Employed part-time	28 (9.2%)
Unemployed	15 (4.9%)
Retired	7 (2.3%)
Self-employed	24 (7.9%)
Stay-at-home-parent	8 (2.6%)
Student	37 (12.1%)
Disabled	4 (1.3%)
Other	2 (0.6%)
*Ethnicity*	
White	264 (87.7%)
Asian	12 (4%)
Black	2 (0.7%)
Latin	17 (5.6%)
Other	6 (2.1%)
*Country of residence*	
USA	153 (50.2%)
UK	40 (13.1%)
Canada	57 (18.7%)
Other	55 (18.0%)
*Religion*	
Agnostic	38 (12.8%)
Atheist	62 (21%)
Buddhist	1 (0.3%)
Catholic	68 (15%)
Hindu	3 (1%)
Jewish	11 (3.7%)
None	104 (35.3%)
Other	8 (2.7%)

	*M (SD)*
Age	36.34 (10.76)
Relationship duration in years	9.91 (8.44)

^a^Percentages do not always add up to 100 due to rounding up

### Measures

The following demographics information was collected from participants: gender, age, sexual orientation, relationship status, relationship length, children, country in which the participant currently lived, education, employment status.

As mentioned previously, the data reported in this paper are part of a larger study and focused on the answers to five open-ended questions about the participants’ own understanding and experiences with sexual need responsiveness. These five questions were (in order of appearance):

1. What does being responsive to someone’s sexual needs mean for you?
2. Describe a time when you felt your partner respected/valued and understood your sexual needs.
3. How do you know what your partner needs sexually?
4. How does your partner know what you need sexually?
5. When you and your partner engage in sexual activity (e.g., kissing, cuddling, touching, oral, penile-vaginal intercourse) how do you meet each other’s needs?

### Analyses

We performed an inductive content analysis on participants’ responses to these open-ended questions (Hsieh & Shannon, 2005). First, the five authors each independently read all the answers to one of the questions, noting down recurring themes. The authors then compared the themes that emerged from this to identify the themes present in all questions. This was converted into a codebook in which we clearly defined all themes (see OSF project page for the full codebook). Then, each author independently coded the answers to one of the questions according to the codebook and double-coded the first 25% of another question to establish interrater reliabilities. Where reliability was insufficient after the first coding, the authors discussed their coding discrepancies and recoded the responses together. Finally, we discussed the codes until 100% agreement was reached on which codes to use. The final kappa’s reflect the reliability after these discussions had taken place and the responses were recoded accordingly (Table 2). The data and materials are available on the Open Science Framework: https://osf.io/rsn7f/

**Table 2:** The inter-rater agreement (kappas) and percentage of participants that mentioned each code for each question

	Kappa	Q1	Q2	Q3	Q4	Q5
Verbal communication						
Conversation	0.68	19.6	20.6	50.6	41.5	34.6
Asking	0.78	6.2	11.4	30.2	19.9	13.7
Telling	0.72	0.7	9.6	15.9	23.5	9.8
Openness and honesty	0.62	4.9	4.9	18.2	18	7.5
Listening						
To verbal cues	0.69	43.5	17.3	24.4	20.9	18.3
To non-verbal cues	0.73	10.1	9.2	14.0	8.8	19
Accommodation	0.67	65.7	41.2	26.3	14.7	13.4
Concession	0.80	0.7	0.7	4.5	0.3	4.6
Respect	0.59	34.3	31.4	17.2	20.3	14.1
Experimentation	0.53	6.5	16.7	11.7	6.2	3.6
Mutual satisfaction	0.63	7.8	12.1	1.6	4.6	29.4
NA	0.5555	3.3	6.2	12.0	15.0	10.8

Communication includes percentages of when the participants only mentioned communication but not asking, being told, or open and honest

## Results

### Definition of Sexual Need Responsiveness (RQ1)

Below we outline the results by briefly describing each theme in turn, providing its definition and some illustrative examples from the dataset. After this, we present the frequencies of the codes for each question. There were a total of seven main themes: verbal communication, listening to verbal and non-verbal cues, accommodation, concession, respect, experimentation, and mutual satisfaction. Some participants indicated they did not have an answer to the open-ended question, did not have sex anymore, or their partners were not responsive (anymore). We coded these responses as not applicable (e.g., “I gave up on this a long time ago”) as these were not relevant for a definition of sexual need responsiveness.

#### Verbal Communication

In the present study, participants identified verbal communication (with subthemes of conversation, asking, telling, and openness and honesty) as an important component of sexual responsiveness. Overall, communication about sex often occurred regularly, sometimes also during sex where it took the form of partners checking in with each other. Communication was mostly aimed at exchanging likes, and dislikes and talking about respective needs. Responses were coded into a subtheme conversation when participants mentioned any form of communication, often implying a back-and-forth conversation. Communication could also happen via directive talk where the participants stated that they and their partner simply told each other what they like, want, or need: “When I told him no more tries at anal sex” (Q2). Other times communication was initiated with a question. Participants reported that they asked each other questions about their likes and dislikes, and also invited each other’s feedback during or shortly after sex: “My partner was asking questions about what I liked and whether his impressions of what I liked were in line with my actual feelings and needs” (Q2). Participants sometimes wrote about the form of their communication about sex as being open and honest. This involved partners creating a safe space to talk, where they could both say what they wanted to say, without judgment from the other party: “Open communication before, during, and after sexual activity” (Q3).

#### Listening to Verbal and Non-Verbal Cues

Communication usually also involves listening, but these themes were distinguished in the study because it is possible to have communication without listening and to listen without deliberately communicating. Listening was reported as an important factor in sexual needs responsiveness by participants, and was divided into two subthemes as partners wrote about listening to each other’s verbal communication (listening to verbal cues) but also about listening to non-verbal cues. The latter involved partners being sensitive to and reading each other’s body language and/or emotional reactions. Sometimes bodily cues took the form of active guiding, sometimes they were more unintentional and more subtle. In essence, participants reported that they and their partner felt each other’s needs rather than having to explicitly communicate about them. For example, one participant said they show sexual need responsiveness “by listening to each other emotionally as well as physically and responding accordingly” (Q5) and another participant said: “we pay attention to signals, talk, and are in touch with our own and each other's body language” (Q5). Listening was also evidenced by partners remembering what they told or showed each other previously. Especially for individuals in a longer relationship, listening culminated in simply knowing each other’s needs and wishes and having these incorporated in routines. These partners were completely in tune, and everything flowed naturally. No explicit communication seemed to be necessary anymore: “we have been in a sexual relationship for nearly 20 years, we are very familiar with each other’s needs” (Q5).

#### Accommodation or Concession

The themes of accommodation and concession are discussed together in this paragraph as they are contrasting ways of managing differences in sexual needs between partners. In situations where participants reported that they and their partners had opposing needs or where one of the partners could not respond in the way requested by the other, participants reported that they tried to find a way to accommodate each other’s needs: They adjusted and made sure that both their needs were taken into account. In this case, the participants stated that they put in effort to respond to each other’s requests happily.^2^ On the other hand, when participants reported that they responded to each other’s requests out of fear of losing or upsetting their partner, this was termed concession. Accommodation involved partners responding to each other’s requests even if it was not their preference because they enjoyed making the partner happy and fostering their connection if they felt comfortable doing so: “Last weekend my partner and I woke up and she wanted to have sex, but I was slower to wake up so I pleasured her for a little while with a toy and then we could move into a more intimate space of love making” (Q2) and “I show this by listening and taking on board what she says about boundaries and desires and doing things she has said or shown that she likes, even if they're not my thing specifically (e.g., she likes being bitten; I don't love doing it but I do it because I know she likes it—and I get pleasure from knowing she is having a good time)” (Q3). Concession involved one partner doing things for their partner not because they wanted to but because their partner wanted it and they wanted to avoid an argument. There was no joy in doing what their partner wanted: “Listening and being open to performing requests which may make me feel silly. The “good giving and game” approach” (Q1).

#### Respect

Participants identified respect as another meaningful component of sexual needs responsiveness. Respect was evident in partners understanding, recognizing, and acknowledging each other’s boundaries, needs, and interests, and behaving in a manner which respected these. It involved holding back when needed and not pressuring each other to do things they would not want. Participants said they tried to understand each other, take each other’s perspective, and empathize with one another. They reported being patient, taking the time for each other, and accepting the partner and their needs, likes, and dislikes. This also involved consent and thereby safety: participants said they and their partner only engaged in activities that they both agreed on, making them feel safe with each other. In this way, partners co-created a safe space of mutual consent. For example, one participant said “I respect when he says not interested and make an effort to get in the mood when he is” (Q3). For some participants, respect involved not only consent and respecting boundaries but also being responsive to a partner’s need of being taken care of and enjoying the process of sex as opposed to focusing on orgasms as an outcome of sex as can be seen from this quote: “Our sex is collaborative, it’s not just about an orgasm it’s about making the other feel taken care of, in a small way “worshipping” them for the duration. We ask for what we want, and if we push boundaries then we do so only after checking in with the other” (Q5).

#### Experimentation

Experimentation was coded as present when the answer included a reference to partners being flexible and willing to change their routines and try out new things. Participants who indicated experimentation as an important aspect of sexual needs responsiveness said they and their partner were curious and receptive; they were willing to consider or accept new suggestions and ideas. This could either happen upon request by the partner or be self-initiated. For example, one participant said “We try sex games and we use them to understand each other’s comfort levels with things we want to try. My partner has items on a hard no list that are on my maybe list and we agreed that if they change their mind we will come back to it” (Q3), another participant said “He asks me. He tries things and makes an effort not to take it personally if they don't work” (Q4).

#### Mutual Satisfaction

The theme mutual satisfaction involved partners making sure that they both enjoyed the sex and ended up satisfied, for example, by making sure they both have an orgasm as can be seen in this quote: “Positive intention, never trying to satisfy just oneself, and look at our sex life as a living thing that requires a certain level of attention that is critical to our emotional and physical health.” (Q5). Ensuring each other’s satisfaction could also involve reciprocity when it was accomplished by partners returning each other’s favors. For example, one participant said: “We both guide the activity towards what we want through guiding touch and talking. if either of us is not/not fully satisfied we try something else to meet remaining needs (oral sex, fingering/hand job on the partner who has not come yet, or masturbation next to partner)” (Q5). Finally, as another participant mentioned, mutual satisfaction could be derived from being focused on the partner and pleasuring them: “Taking pleasure in your partner's pleasure.” (Q1) which implies that responsiveness to sexual needs is a relational characteristic.

### Frequency of Themes (RQ2 and RQ3)

Table 2 shows per question the percentages of participants that mentioned each theme. Most participants included several themes in their answers which together created their definition of sexual need responsiveness and indicated their behaviors in their sexual relationship. Some of the questions lent themselves more to certain types of codes: specifically Question 1 concerned the definition of sexual need responsiveness and Questions 3, 4, and 5 asked about responsive behaviors. Question 2 was in-between by asking about a specific time when the participant had felt their partner understood and valued their sexual needs. Consequently, the more abstract codes were relatively frequent in Question 1 and the behavioral codes were relatively infrequent, compared to the other questions. However, there was a large amount of overlap in the themes across the five questions suggesting that the definition and execution of sexual need responsiveness are broadly similar.

Q1 explicitly asked participants to define what sexual need responsiveness meant for them. The question had a high prevalence of accommodation (65.7%), respect (34.3%), and listening (43.5%). Participants tended to define sexual need responsiveness as accommodating each other's needs and treating each other with respect. In other words, attempting to take both partners’ needs into account while respecting the boundaries of each other. Listening was one clearly behavioral theme which occurred frequently in Q1. Conversation (19.6%) was mentioned half as often as listening (43.5%), which suggests that the listening that defines responsiveness does not necessarily mean having a conversation. Thus, sexual need responsiveness may be more about listening to verbal or non-verbal cues from a partner rather than having a conversation as such about needs and desires. Perhaps it is because, to be responsive, in other words to understand, to accept, and to value the partner, one first needs to learn about the partner’s needs and preferences. Learning about the partner's needs and preferences is done through listening, both at what is said verbally and shown non-verbally. The rest of the themes were infrequent within participants’ definitions of sexual need responsiveness. In short, the definition of responsiveness appeared to revolve around partners taking a step back by adapting (happily) to each other's needs, acknowledging and not crossing each other’s boundaries, and listening to what their partner had to say.

When asked to think back to a time when the participants felt their partner was responsive (Q2), participants’ responses included a high prevalence of accommodation (41.2%) and respect (31.4%). Conversation (20.6%) and listening (17.3%) were mentioned with similar frequency. Compared to the other questions, experimentation (16.7%) was the most frequently mentioned in the answers to this question. Thus, the participants’ descriptions of a responsive time were similar to their definition of responsiveness but there was more heterogeneity (i.e., different people mentioned different themes) in themes compared to Q1 and responsiveness was often experienced in times of sexual experimentation.

Verbal communication appeared to be key in knowing what one’s partner needs (Q3) and letting one’s partner know what one needs (Q4). To learn about their partner's needs and to teach their partner about their needs, participants talked to each other, asked questions, were asked questions, or just told each other what they wanted. Participants were more likely to say that they would ask about their partner’s needs in Q3 (30.2%) compared to in Q4 (19.9%). In contrast, they were more likely to say that they would tell their partner their needs in Q4 (23.5%) compared to in Q3 (15.9%). However, this may just reflect a matter of perspective as the way the questions were asked could have led the participants to respond in terms of their own behavior and not their partner’s behavior (Q3: “How do you know what your partner needs sexually?” = I ask them; Q4: “How does your partner know what you need sexually?” = I tell them). The codes asking and telling were much more frequent in these questions compared to the first two questions given that Q3 and Q4 focused more on explicit behaviors and ways in which sexual need responsiveness was communicated rather than its definition. Listening was mentioned around half as often as verbal communication. This may reflect the way the questions were phrased as listening is a more passive activity whereas the questions asked about what was actually done. Furthermore, in Q3, participants wrote relatively frequently about accommodation (26.7%), suggesting that partners also acted to satisfy each other’s needs rather than solely discussing about them. Respect was mentioned by one fifth of the participants in both questions (Q3 and Q4) which was less frequent compared to the first two questions. This may be because respect is a relatively abstract term that participants do not think of when asked how they know what their partner needs or how they let their partner know about their needs.

Q5 focused on how partners met each other’s needs when they were engaged in sexual activity. The codes conversation (34.6%) and mutual satisfaction (29.4%) were the most frequently mentioned codes within this question. Body sensitivity and listening were mentioned by one fifth of the participants in Q5. Partners thus met each other’s needs during sex by listening and attending to each other’s (intentional and unintentional) bodily cues and verbally communicating when needed. One third of the participants mentioned mutual satisfaction being an important aspect of actually meeting each other’s needs whereas in other questions the theme only arose infrequently. It may be that mutual satisfaction is considered a measure of success of being sexually responsive in a sexual scenario; the outcome of responsiveness. Accommodation and respect were mentioned less frequently compared to in other questions but were still present about 15% of the time. Thus, during sexual activity, partners are responsive by accommodating or stepping back to meet each other’s needs as well.

Across all questions, concession was the only theme which was mentioned less than 5% of the time. Concession implies giving something away and one partner being dissatisfied with the outcome. In other words, forced sacrifice. Accommodation, on the other hand, implies that both partners’ needs are being taken into consideration and met as much as possible. Accommodation was mentioned between 13.4 and 65.7% of the time suggesting that accommodation is a more representative theme of sexual need responsiveness compared to concession.^3^

## Discussion

The aim of this study was to develop a participant-informed definition of sexual need responsiveness. We performed a content analysis on participants’ answers to open-ended questions about their understanding of the concept and how they themselves experienced and enacted sexual need responsiveness in their relationship. Based on this, we define sexual need responsiveness as: “being willing to communicate and listen to a partner’s verbal and non-verbal cues of what they want and need sexually and accommodating these wants and needs while retaining the autonomy to make individual decisions, free from pressure, shame, or guilt.” This definition emphasizes accommodation of both partner’s needs (cf. perceived partner responsiveness) rather than concession (cf. unmitigated sexual communion). Our definition also incorporates consent, listening, and communication, which have not been a part of existing definitions of sexual need responsiveness. Below, we provide a more detailed description of each of the themes and how they relate to the definition and previous literature.

Accommodation referred to trying to accommodate each other’s needs, adjusting when unable to respond, and making sure that both partners’ needs were considered. This construct is different from the definition of sexual need responsiveness as sexual communal strength, which would suggest being motivated to meet a partner’s needs without expecting this to be reciprocated (Muise et al., 2013) rather than accommodating both partner’s needs. Furthermore, concession only occurred in under 5% of the responses throughout the questions suggesting participants did not perceive unmitigated sexual communion as an inherent part of sexual need responsiveness. Indeed, previous research has found that unmitigated sexual communion can be detrimental to sexual and relationship satisfaction (Impett et al., 2019). Thus, it seems important that partners are equal in sexual relationships and try to accommodate each other’s needs but not at the expense of the self.

Respect was also an important element of sexual need responsiveness which was defined as understanding, recognizing, and acknowledging each other’s boundaries and behaving in a way that respects these boundaries. The respect element of the definition was closest to what we traditionally define as perceived partner responsiveness (Reis et al., 2004) which involves understanding, validating, and caring about a partner’s needs. Perceived partner responsiveness has been shown to be associated with sexual and relationship satisfaction (Rosen et al., 2020) and sexual desire (Birnbaum & Reis, 2012; Birnbaum et al., 2016; van Lankveld et al., 2021). Furthermore, it was clear in the participants’ responses that consent was a prerequisite of being responsive to a partner’s sexual needs, which is not a part of a traditional definition of perceived partner responsiveness or sexual communal strength. Respect is a factor contributing to relationship success and involves taking responsibility for obtaining a partner's consent before and during sexual activity. In other words, for there to be respect, all sexual activity must be consensual with both partners, who should constantly check the partner's consent and express their own (i.e., ask or tell again openly and honestly if things are not clear; Owen et al., 2012). Sexual consent has received a great deal of attention in the academic literature, especially following movements such as #MeToo (e.g., Jozkowski et al., 2014a, 2014b, 2017; Mark & Vowels, 2020) and it was also clear in the present study that consent was an integral element of respecting one’s partner.

Furthermore, verbal communication and listening were also considered important elements of being sexually responsive. Communication involved both reciprocal conversation (i.e., mutualistic sexual talk) and unidirectional expression of wishes and desires (i.e., individualistic sexual talk) in an open and honest manner. Listening, on the other hand, included being attentive to both verbal and non-verbal cues for what the partner wanted sexually. Definitions of sexual communal strength (Muise et al., 2013) and perceived partner responsiveness (Reis et al., 2004) do not explicitly incorporate communication or listening as a part of their definition but participants in the present study considered both the expression of needs and desires as well as listening to a partner’s expression of them as integral to sexual need responsiveness. Previous research has found that perceived partner responsiveness was important in buffering the potential negative effects of individualistic talk on sexual and relationship satisfaction (Merwin & Rosen, 2020). The present study goes beyond this research to suggest that communication and listening should be incorporated into a definition of sexual need responsiveness.

Finally, the themes experimentation and mutual satisfaction were mentioned less often than the aforementioned themes when participants were explicitly asked to provide their definition of sexual need responsiveness (6.5% and 7.8%, respectively). However, more participants talked about experimentation when asked about behaviors indicating sexual need responsiveness. Engaging in novel and interesting activities together with a romantic partner (i.e., self-expansion) has previously been shown to improve sexual desire and relationship satisfaction in long-term relationships (Muise et al., 2018a, 2018b, 2019; Raposo et al., 2020). The present research suggests that sexual self-expansion via experimentation can be seen as a part of being responsive to a partner’s sexual needs. Indeed, experimentation involved trying new things with a partner that could enhance each partner’s enjoyment of sex and to keep things interesting for both partners. Mutual satisfaction, on the other hand, primarily arose when describing an outcome of partners being responsive to each other’s sexual needs. Indeed, sexual satisfaction has often been considered as an outcome of perceived partner responsiveness (Rosen et al., 2020) or sexual communal strength (Balzarini et al., 2021; Day et al., 2015; Hogue et al., 2019) and the present study showed that a third of the participants explicitly considered mutual satisfaction as an important outcome of being sexually responsive. We did not incorporate experimentation or mutual satisfaction into our definition because these themes were less often described by participants as integral to the definition. Instead, we expect experimentation to be a part of accommodation for couples who see experimentation as an integral part of their sexual expression.

### Definition vs. Behavior

In addition to providing a participant-informed definition of sexual need responsiveness, the present study also aimed to understand how the definition of sexual need responsiveness compares to how people enact and perceive it being enacted in their relationships, that is, responsive behavior. When participants were asked about a time when they felt their partner was responsive to their sexual needs, many participants described their partner accommodating and respecting their needs but also talked about communication and listening. Thus, when participants were asked about when they felt responded to, this was in line with their definition of sexual need responsiveness. Explicitly communicating about sexual needs was an important element of how partners were able to be sexually responsive to each other, which could be done either by having a conversation, through asking, or by telling one’s partner about what one needs in an open and honest way. This finding is in line with previous research which has found that communication is an important element of successful and satisfying sexual relationships (Hansson & Ahlborg, 2016; Leistner & Mark, 2020; Mark & Jozkowski, 2013; Shapiro et al., 2000). Sexually responsive behaviors also involved listening and respecting each other’s needs. During sex (Q5), participants also valued both verbal and non-verbal communication but often measured the success in being sexually responsive by whether they and/or their partner was sexually satisfied at the end of the sexual encounter. Indeed, mutual satisfaction seemed to only be the metric against which participants measured whether they had been sexually responsive to their partner, not an integral part of the definition of sexual need responsiveness.

### Implications for Theory and Practice

The study makes a novel contribution to the theory of sexual need responsiveness by providing a participant-informed definition of sexual need responsiveness in relationships. It adds to the previous definitions of sexual need responsiveness (Impett et al., 2020) by highlighting the importance of mutuality and respect for boundaries. We found that participants viewed accommodation of both partner’s needs important rather than prioritizing one person’s needs over another person’s needs (unmitigated sexual communion). The definition of perceived partner responsiveness (Reis et al., 2004) does not involve respect specifically for boundaries. However, given the importance of consent in a sexual scenario (e.g., Jozkowski et al., 2014a, 2014b, 2017; Mark & Vowels, 2020), respect for boundaries is likely to be one of the most important differences between general responsiveness in relationships and sexual need responsiveness. The participant-informed definition also highlights understanding and respect toward a partner’s needs even when those needs cannot be met. This suggests that being responsive to sexual needs involves accommodating each other’s needs when possible but even more importantly, understanding, and respecting one’s needs regardless of whether these needs can be met.

The study also has some important practical implications for clinicians working with couples and sexual relationships. Given conflicts about sex are among the most difficult problems to solve (Geiss & O’Leary, 1981; Sanford, 2003) but are also more predictive of interpersonal vulnerability (Rehman et al., 2017, 2019) and relationship quality (Rehman et al., 2017) than non-sexual conflicts in relationships, it is important for clinicians to address sexual need responsiveness in therapy. While some therapists are likely to already address this issue in therapy, many couples therapists feel ill-equipped to discuss sexuality with couples (Hipp & Carlson, 2019). Based on the findings, we advise clinicians to have a conversation about how partners can accommodate each other’s needs in a sexual scenario, help couples communicate and listen to each other’s needs, and to be mindful of respecting each other’s boundaries. It can also be important to understand both the expectations of what sexual need responsiveness means for each partner as well as the actual behaviors in a relationship given that the results from the present study showed that, while there is overlap, perceived definition and actual behavior may not be the same. It is possible that if one’s partner’s behavior falls short from one’s expectations, one may become unsatisfied.

### Strengths and Limitations

The present study had several strengths including the use of open-ended questions which enabled participants to provide their own definition of sexual need responsiveness rather than choosing from a ready-made selection of definitions from literature or being provided with a definition. The survey also allowed us to hear from a larger number of people than interviews would have made possible. The definition was informed by participants rather than it being expert-led. We also asked participants different questions to gain a more comprehensive understanding of the definition of sexual need responsiveness and how participants’ behaviors compare to this definition. This added to our understanding of the definition and made it more generalizable than relying on one specific question.

There were also several limitations to this study that should be considered. Although open-ended responses allowed us to gather data from many participants and for participants to come up with their own ideas, it is also possible that they did not think about something that they would consider responsive. We tried to mitigate this by asking them multiple questions that could help remind them of what they thought of as responsiveness. However, multiple similar questions could also have led to participants becoming bored or annoyed, consequently deteriorating the validity of the responses to the later questions. We also could not go as in-depth as interviews would have allowed. The participants had also already responded to some questionnaires that included questions about responsiveness. Thus, the participants were not entirely naive to the expert definition of sexual need responsiveness. However, some themes, such as consent, were not mentioned in the questionnaires and thus this was important to participants regardless of what they were presented with before. Furthermore, some of the inter-rater reliabilities were weak primarily due to having only a small number of observations within the 25% that were double coded.

The sample also consisted primarily of a convenience sample of mostly women from English-speaking Western societies, and thus, the definition may be less accurate for men or people from different cultures. Although there was no difference in sociodemographic characteristics between participants who responded to the open-ended questions compared to those who did not, it is important to note that participants with greater sexual experience and more positive sexual attitudes tend to volunteer for sex-related studies (Dawson et al., 2019). This may have been the case in our study as well and could have impacted obtained results: For example, given the participants were responding to a study about sex, they may have been having higher quality sex and been more responsive than the average population, focused more on experimentation and mutual satisfaction. Additionally, consent and boundaries may have been over-represented as consent education has become increasingly common in many Western societies. Finally, some of the questions lent themselves to different types of themes (behavior vs. definition) and thus they were not directly comparable although the high degree of similarity in the themes across the questions indicates cohesion in the definition.

### Future Directions

The present study provides several directions for future research. Currently, sexual need responsiveness has primarily been examined from the communal strength perspective using the sexual communal strength scale (for recent reviews, see Impett et al., 2015, 2020; Muise & Impett, 2016). However, the scale does not directly address many of the elements of sexual need responsiveness highlighted by the participants in the present study. It also taps into an individual difference rather than a perception of a partner’s responsiveness. Thus, to understand both perceived and provided sexual need responsiveness in relationships, we need a scale that will incorporate the elements highlighted in this study.

It will also be important to understand whether sexual communal strength and sexual need responsiveness are, in fact, distinct constructs; and whether sexual need responsiveness predicts individual and relational outcomes over and above sexual communal strength. Future research is also needed to validate the definition from this study. One way of doing this could be to present participants with different definitions or asking them to rank the themes that were found in the study in order of importance, or simply asking them which aspects they consider important for being sexually responsive. Moreover, listening to non-verbal cues could be interpreted differently depending on the stage of the relationship. For example, in longer-term relationships, no longer needing to verbally communicate your sexual desires could potentially also be reflective of perceived stability of sexual needs over time, rather than a particularly astute sexual partner. As such, future research should interrogate potential differences in how people define responsiveness over the course of relationships.

The definition also needs to be validated in other samples given that most of the study participants were women, in mixed-sex relationships, and western. Dyadic data would also allow for a comparison between partners’ definitions of sexual need responsiveness to understand whether two partners define responsiveness in a similar manner and whether this has implications for their sexual relationship. This could provide insights for clinicians working with couples to help them navigate potential discrepancies in sexual need responsiveness between partners.

### Conclusion

In summary, the present study provided a participant-informed definition of sexual need responsiveness, which included communication, listening to, and accommodating each other's needs and wishes, and acting on those needs and wishes whenever possible, while respecting both partners’ safety and boundaries. Communicating openly and honestly and listening to both verbal and non-verbal cues was an important way to establish sexual needs in relationships and enabled partners to be sexually responsive. Mutual satisfaction seemed to be used as a measure of success in being responsive. The study extended our definition and understanding of sexual needs responsiveness and highlighted the benefits of asking participants directly about what our constructs mean for them. This can add additional insights for researchers and also highlight areas for clinicians to focus on.

## Footnotes

1 To assess possible differences between participants who responded to the open-ended questions from those who did not, a multivariable logistic regression analysis was carried out with a binary outcome of having responded to the open-ended questions or not. The predictors that were included in the logistic regression analyses were age, gender, sexual orientation, current relationship status, duration of the relationship, having children, highest level of completed formal education, importance of valuing and understanding partner’s needs, importance one puts on their partner valuing and understanding their needs, relationship satisfaction, sexual satisfaction, emotional satisfaction, health functioning, and importance of sex. The participants that were removed from the dataset due to incomplete data, did not significantly differ in any of the demographic or relational characteristics mentioned from those who answered all open-ended questions (AOR_age_ = 1.01, *p* = .35; AOR_relationship length_ = .99, *p* = .41; AOR_education_ = 1.10, *p* = .36; AOR_relationship status_ = .92, *p* = 44; AOR_children_ = 1.36, *p* = .16; AOR_sexual orientation_ = 1.30, *p* = 17; AOR_gender (women—men)_ = 1.05, *p* = .82; AOR_gender (other—men)_ = 0.63, *p* = .502; AOR_importance_self_ = 0.17, *p* = .19; AOR_importance_partner_ = 0.24, *p* = .84; AOR_relationship satisfaction_ = 0.01, *p* = .95; AOR_sexual satisfaction_ = -0.17, *p* = .73; AOR_emotional satisfaction_ = -0.32, *p* = .62; AOR_health functioning_ = 0.23, *p* = .60; AOR_importance of sex_ = -0.78, *p* = .051). For more information, please see the Supplemental Material on the OSF project page, Appendix 1.

2 This theme was initially divided into two constructs ( and accommodation) that were coded independently but they were merged in the description due to extensive overlap. involved trying to find a solution that is acceptable for both partners, making sure both parties’ needs are considered.

3 There were no significant gender differences in the frequency of themes, with two exceptions. For the second question in which we asked participants to describe sexual need responsiveness, there were differences among men and women in the prevalence of two themes, respect and satisfaction, respectively. Women described responsiveness through respect more often than men (38.2% women, 8.9% men; *χ*^2^[2, *N* = 286] = 14.58, df = 1, *p* < .001). However, the association between these two variables was weak (*φ* = − .23, *p* < .001). Women also described responsiveness through satisfaction more often than men (14.9% women, 2.2% men; *χ*^*2*^[2, *N* = 286] = 5.44, df = 1, *p* = .02); however, this association was very weak (*φ* = − .14, *p* = .02). For the fourth question asking how participants’ partners know what participants need sexually, we have found no significant association between the occurrence of the themes and identifying as a man or a woman. However, we have found a significant association between gender and concession (*φ* = .14, *p* = .03; see OSF project page, Supplemental Material, Appendix 2).

## Declarations

### Availability of Data and Material

https://osf.io/rsn7f/?view_only=97248900efa14ccd8e77dd3c62aac2b7.
